# Novel plant inputs influencing *Ralstonia solanacearum* during infection

**DOI:** 10.3389/fmicb.2013.00349

**Published:** 2013-11-20

**Authors:** A. Paola Zuluaga, Marina Puigvert, Marc Valls

**Affiliations:** ^1^Departament de Genètica, Universitat de BarcelonaBarcelona, Spain; ^2^Centre for Research in Agricultural Genomics (CSIC-IRTA-UB-UAB)Bellaterra, Spain

**Keywords:** *R. solanacearum in planta*, plant inputs in hrp regulon, apoplast and xylem contents, novel induction of HrpG, pathogenicity mutants, sugars and aminoacids tomato fluids

## Abstract

*Ralstonia solanacearum* is a soil and water-borne pathogen that can infect a wide range of plants and cause the devastating bacterial wilt disease. To successfully colonize a host, *R. solanacearum* requires the type III secretion system (T3SS), which delivers bacterial effector proteins inside the plant cells. HrpG is a central transcriptional regulator that drives the expression of the T3SS and other virulence determinants. *hrpG* transcription is highly induced upon plant cell contact and its product is also post-transcriptionally activated by metabolic signals present when bacteria are grown in minimal medium (MM). Here, we describe a transcriptional induction of *hrpG* at early stages of bacterial co-culture with plant cells that caused overexpression of the downstream T3SS effector genes. This induction was maintained in a strain devoid of *prhA*, the outer membrane receptor that senses bacterial contact with plant cells, demonstrating that this is a response to an unknown signal. Induction was unaffected after disruption of the known *R. solanacearum* pathogenicity regulators, indicating that it is controlled by a non-described system. Moreover, plant contact-independent signals are also important *in planta*, as shown by the *hrpG* induction triggered by apoplastic and xylem extracts. We also found that none of the amino acids or sugars present in the apoplast and xylem saps studied correlated with *hrp*G induction. This suggests that a small molecule or an environmental condition is responsible for the T3SS gene expression inside the plants. Our results also highlight the abundance and diversity of possible carbon, nitrogen and energy sources likely used by *R. solanacearum* during growth *in planta*.

## Introduction

The life cycle of most bacterial plant pathogens includes a long phase of survival or multiplication in the environment, entry and colonization of plants, and high multiplication in specific plant tissues that leads to symptom development in susceptible hosts. The bacterium successfully adapts to these disparate niches through differential gene expression in response to specific environmental signals (Mole et al., 2007; Saha and Lindeberg, 2013).

*Ralstonia solanacearum* is a soil-borne pathogen that infects more than 200 host species from over 50 botanical families (Peeters et al., 2013). Nonetheless, this pathogen can live as a saprophyte in the soil when there are no hosts available (Schell, 2000; Mansfield et al., 2012). In order to deal with the physiological demands of these contrasting situations, the bacterium possesses a complex regulatory network that responds to both environmental and internal cues (Schell, 2000; Genin and Denny, 2012). The main pathogenicity determinant in *R. solanacearum* is the type III secretion system (T3SS), which translocates effector proteins into the plant host cells (Coll and Valls, 2013). The T3SS is encoded by the *hrp* gene cluster and is regulated by plant and metabolic signals (Brito et al., 1999; Aldon et al., 2000). Plant signals are sensed by the outer membrane receptor PrhA, which responds to still-unknown cell wall components (Aldon et al., 2000) and transduces the signal through PrhR, PrhI, and PrhJ to induce the central *hrp*G regulator (Brito et al., 1999, 2002). HrpG controls the downstream HrpB activator that regulates transcription of the *hrp* genes and related T3SS effectors (Brito et al., 1999, 2002; Valls et al., 2006; Genin and Denny, 2012). Metabolic signals are also sensed in this complex regulatory network by PrhG, a close paralog of HrpG. PrhG is responsible for activating *hrpB* in response to plant cell contact (Plener et al., 2010). HrpG has been proposed to be a master regulator playing a role in the transition from saprophytic to parasitic life style by integrating the plant cell contact signal (Aldon et al., 2000), the metabolic inputs triggered in minimal medium (MM) (Brito et al., 1999), and a quorum sensing signal through the PhcA regulator (Genin et al., 2005). As a result, HrpG co-regulates the induction of the T3SS and other virulence determinants (Valls et al., 2006). PhcA is a global density-dependent regulator that indirectly suppresses *hrpB* expression by either lowering *prhIR* transcription (Genin et al., 2005; Yoshimochi et al., 2009a) or repressing *hrpG* (Yoshimochi et al., 2009b). It was recently reported that *prh*G is activated by PhcA, proposing that *R. solanacearum* switches from HrpG to PrhG to ensure *hrpB* activation in a cell density-dependent manner (Zhang et al., 2013). On the other hand, PhcA controls exopolysaccharide production via XpsR, motility and cell wall-degrading activities via pehSR and quorum sensing through the transcriptional activator solR, responsible of sensing the acyl-homoserine-lactone (AHL) (Brito et al., 1999; Aldon et al., 2000; Genin et al., 2005).

Despite the wealth of knowledge gained in the last years, we remain quite naïve about the environmental inputs that elicit the *R. solanacearum hrp* regulon in natural conditions, as most gene regulation studies have been performed in *in vitro* culture. Discrepancies with recent gene expression analyses *in planta* (Monteiro et al., 2012) suggest that the bacterium may receive unknown signals during saprophytic life. In this work we aimed to further our understanding of the environmental inputs that control the *hrp* regulon and identified a novel regulatory signal triggering *hrpG* expression at early stages of the interaction with *Arabidopsis* cells. We demonstrate that this signal is not dependent on PrhA or PrhJ, and that, contrary to what was currently known, *hrpG* can be strongly activated by plant apoplast and xylem saps in the absence of cell-wall derived signals. We describe the most abundant carbon and nitrogen sources available *in planta* for pathogen growth and conclude that none of them seems to influence the newly described *hrpG* induction.

## Results and discussion

### The hrpg regulon is induced by a cell contact-independent signal when *R. solanacearum* grows in the presence of plant cells

**Figure 1:**
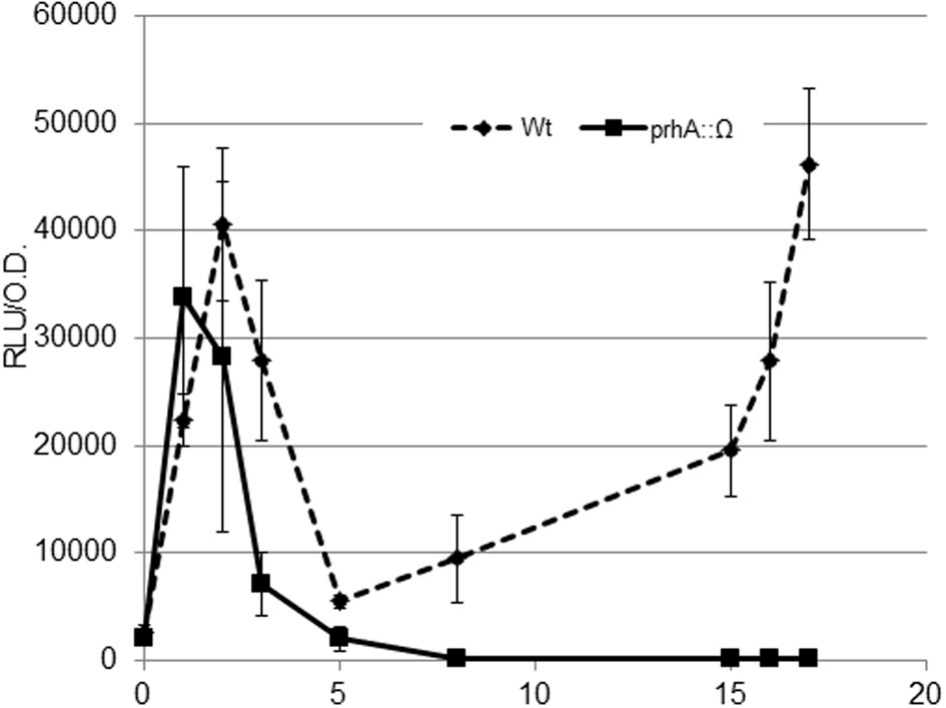
**Expression profile of the *hrpG* promoter during bacterial co-culture with *Arabidopsis* cells.** The wild-type *R. solanacearum* strain GMI1000 (wt, solid line) or its *prhA*-defficient derivative (prhA::Ω, dashed line) carrying the *PhrpG::LuxCDABE* fusion were grown in Gamborg medium in the presence of plant cells and luminescence measured at different time points. A representative result using three biological replicates is shown. Promoter output is presented as relative luminescence units (R.L.U) produced by the *lux* reporter corrected by cell density estimated by OD_600_. Error bars indicate standard deviations.

*HrpG* is a central transcriptional regulator that drives the expression of the T3SS and other virulence determinants in *R. solanacearum* (Genin and Denny, 2012; Zhang et al., 2013). Studies on *hrpG* expression have often been carried out measuring transcriptional output from cultures grown overnight in MM or in co-culture with plant cells (Marenda et al., 1998). In order to gain a better understanding of *hrp* regulation earlier in the plant-pathogen interaction, we performed a time course experiment in co-cultures of *R. solanacearum* with *Arabidopsis* cells by measuring transcription of *hrpG* every hour. To this end, we used a modified bacterial strain containing the *hrpG* promoter fused to the *luxCDABE* operon integrated in a permissive site of the chromosome (Monteiro et al., 2012). With this strain, real time information of *hrpG* transcription could be obtained by measuring luminescence. To our surprise, the wild type (wt) strain showed a bimodal induction of *hrpG*, with clear induction of the *HrpG* promoter during the first 2 h of co-culture with plant cells (Figure 1). This early induction was significant, although less strong than the one observed at later times (8–16 h). PrhA is the outer membrane receptor responsible for the well-described *hrp* gene induction upon contact with plant cells (Marenda et al., 1998). To check if the two *hrpG* induction peaks observed were mediated by PrhA, we introduced the *PhrpG::lux* construct in the *prhA* mutant background and evaluated luminescence. Interestingly, the strong induction at 2 h of co-culture with plant cells was maintained while the second peak was abolished (Figure 1). Thus, the outer membrane receptor PrhA mediated the second induction peak but was dispensable for *hrp* induction at early stages. This was confirmed by the fact that the early induction also remained unaltered in a strain deficient for PrhJ, the regulator that transduces the PrhA cell-contact induction to HrpG (data not shown). In these experiments, high variability in gene expression was observed at early times, likely due to the fact that cultures were still adapting to the new growth conditions in co-culture after dilution. However, a robust induction resulting in the early expression peak was always detected.

**Figure 2:**
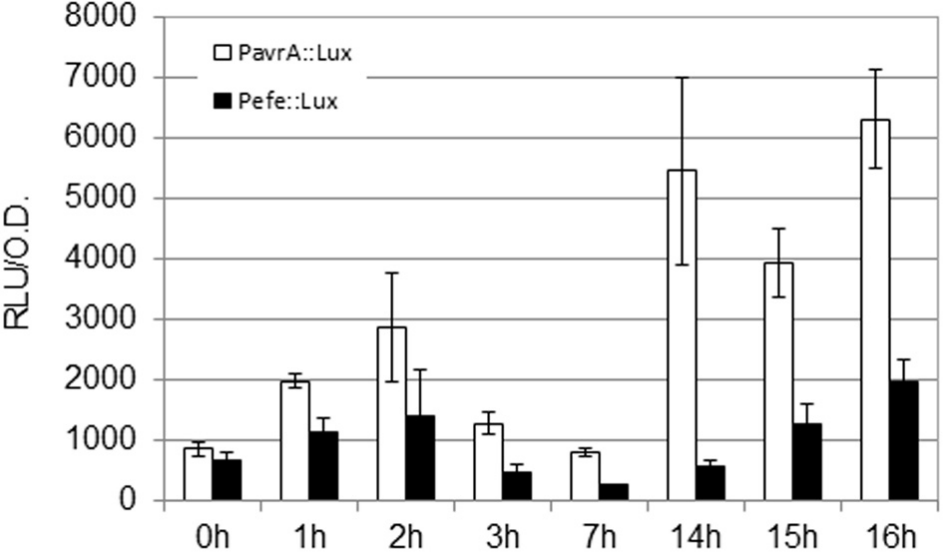
**Expression profile of *HrpG*-controlled genes in co-culture with *Arabidopsis* cells.** Luminescence was measured from *R. solanacearum* GMI1000 carrying fusions of the *avrA* or *efe* promoters to the *luxCDABE* reporter (PavrA-lux or Pefe-lux, respectively). Growth conditions and units as in Figure 1.

In order to test the relevance of the initial *hrpG* induction on downstream genes, we measured the expression profiles of the type III-secreted effector *avrA* and the gene coding for the *R. solanacearum* ethylene-forming enzyme (*efe*). These genes were selected because *avrA is* controlled by HrpG and HrpB while the *efe* gene is specifically regulated by HrpG in a HrpB-independent manner (Valls et al., 2006). The *R. solanacearum* PavrA-lux and Pefe-lux strains were created and luminescence was measured as shown in Figure 2. Absolute expression levels of the *avrA, efe*, and *hrpG* promoters differ due to different promoter strength, but remarkably, expression of both downstream genes showed the bimodal profile, maintaining an early induction at 2 h of co-culture with plant cells (Figure 2). Differences in the magnitude of the early induction in these genes might be explained by additional regulatory inputs that over impose to the HrpG action. We presume that this induction could have implications in plant-pathogen interactions, since pathogen effectors are being expressed and likely secreted to the host within few hours of contact with the host cells.

### The PrhA-independent induction of *HrpG* is not mediated by any of the known pathogenicity regulators

**Figure 3:**
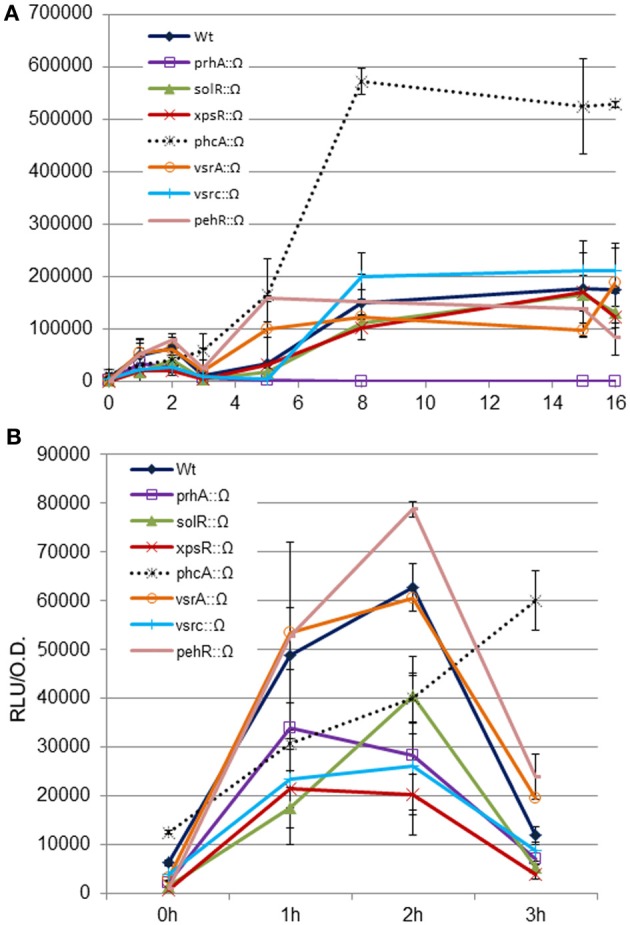
**Expression profiles of the *hrpG* promoter in wild type *R. solanacearum* or virulence regulatory mutants in co-culture with Arabidopsis cells.** The wt or the prhA, solR, xpsR, phcA, vsrA, vsrC, and pehR disruption mutants carrying the *PhrpG::LuxCDABE* fusion were grown in Gamborg medium in the presence of plant cells and luminescence measured at different time points **(A)**. Zoom-in of *hrpG* expression during the first 3 h of co-culture with *Arabidopsis* cells to better appreciate that all mutants show comparable expression profiles as the wild type in the first induction peak during growth in the presence of plant cells **(B)**. A representative result using three biological replicates is shown. Promoter output is presented as relative luminescence units (R.L.U) produced by the *lux* reporter corrected by cell density estimated by OD_600_. Error bars indicate standard deviations.

Next, we used a classical genetics approach to determine if any of the known regulatory pathways mediated the newly-identified signal. To this end, we tested *hrpG* induction in *R. solanacearum* mutants defective in the main pathogenicity regulators (Supplementary Table 1). The analyzed mutants were: *solR*, a quorum sensing transcriptional activator responsible of sensing the AHL; *vsrA*, which activates *xpsR* and represses motility; *vsrC*, an activator of EPS and motility that represses pectinase activity; *xpsR*, which induces EPS synthesis; *phcA*, the master regulator that represses *hrpG* in culture, and *pehR*, which activates the pectinase-encoding gene *pehA* and motility (Genin and Denny, 2012). All these mutants were transformed with the *hrpG* promoter fused to the lux operon and luminescence measured in co-culture with plant cells. Figure 3A shows the expression profiles in the wt or the *phcA*, *pehR*, *vsrC*, *solR*, *xpsR*, and *vsrA* mutants. All mutants showed a similar expression profile, except for *phcA*, that showed a strongly enhanced *hrpG* expression, as expected due to the well-described PhcA inhibition on *hrp* gene expression (Genin et al., 2005). Figure 3B shows a zoom-in of the *hrpG* expression profile during the first 3 h of co-culture. All the regulatory mutants maintained the early cell-contact-independent induction of *hrpG*, indicating that none of them is directly mediating the inducing signal. This finding suggests that there are yet unknown triggers of *hrp* gene expression.

### Plant cell contact is not essential for *HrpG* induction *in planta*

**Figure 4:**
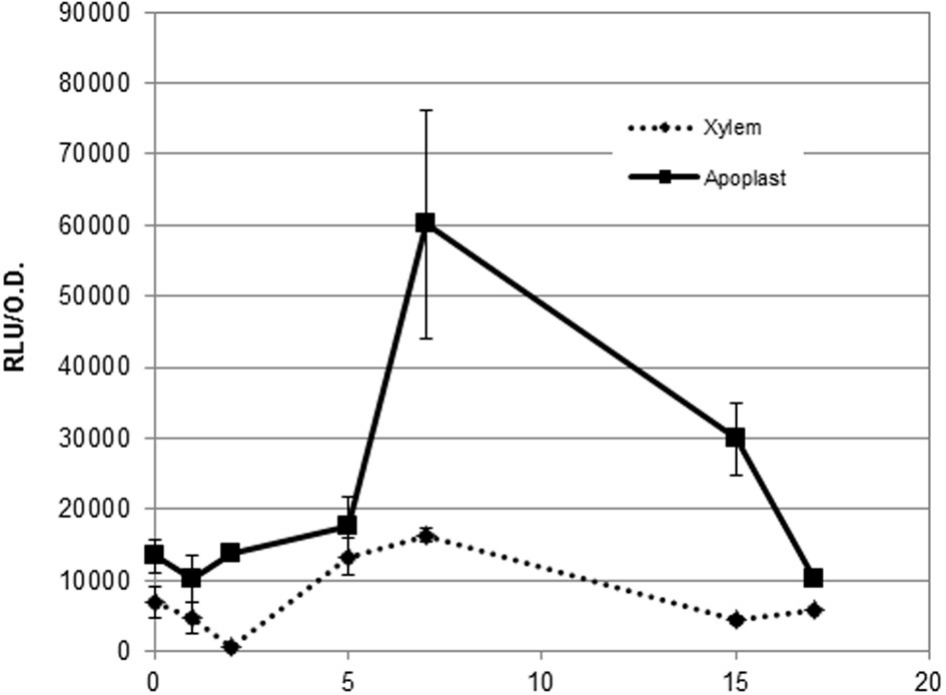
**Expression profile of the hrpG promoter in both apoplast and xylem exudates.** Induction of the hrpG promoter in bacteria grown in cell-free plant exudates. A representative result using three biological replicates is shown. Promoter output is presented as relative luminescence units (R.L.U) produced by the *lux* reporter corrected by cell density estimated by OD_600_. Error bars indicate standard deviations.

To test whether plant cell contact-independent induction also occurs *in planta*, we extracted apoplast and xylem fluids from tomato plants as described in (Coplin et al., 1974; Rico and Preston, 2008) and performed *hrpG* expression profiles from bacteria growing in both exudates (Figure 4). In agreement with our previous observations, we observed high expression of the *HrpG* promoter comparable to the induction we detected in co-cultures (Figure 3) when bacteria were grown in plant extracts in the absence of plant cells (Figure 4). However, differences in growth rate and bacterial physiology in plant extracts and co-cultures with plant cells make it difficult to compare the induction timings in both conditions. These results indicate that, contrary to what has been hypothesized until now, cell contact-independent induction of *R. solanacearum hrpG* is also triggered *in planta* in addition to the well-established cell wall contact response mediated by PrhA (Aldon et al., 2000; Brencic and Winans, 2005; Rico and Preston, 2008). The precise plant molecules or environmental cues responsible for the newly described *hrp* gene induction remained unknown.

### No aminoacid or sugar in tomato extracts correlates with *HrpG* expression

To shed light on the nature of the signals involved in contact-independent *hrp* gene induction *in planta*, we performed an analysis of the sugars and amino acids present in both tomato xylem sap and apoplastic fluid before and after sustaining growth of *R. solanacearum*. These fluids were chosen because they correspond to the two main compartments where *R. solanacearum* grows inside the plant (Vasse et al., 1995; Ward et al., 2010). Since the observed *hrpG* induction peaked at 7 h of growth in the apoplastic fluid but remained roughly unchanged in the xylem, we searched for an aminoacid or sugar whose abundance diminished after 7 h of *R. solanacearum* growth in apoplast sap and that showed a similar pattern or remained constant in xylem cultures. Molecule contents for both xylem and apoplast were measured by chromatography at time 0 and at 7 or 21 h after bacterial growth and are presented in Tables 1, 2. Surprisingly, sugars and aminoacids concentrations were similar at either 7 or 21 h of bacterial growth; thus, only the latter time point is shown. However, none of the detected molecules showed the expected abundance profile, indicating that common aminoacids or sugars do not play a role in the induction of *hrpG* and the subsequent trigger of the main *R. solanacearum* virulence determinants *in planta*.

**Table 1:** **Sugar content in tomato exudates before and after growth of *R. solanacearum***.

	**Apoplast**		**Xylem**	
	**− bacteria**	**+ bacteria**	**− bacteria**	**+ bacteria**
Sucrose	193.43	21.22	ND	ND
Fructose	156.74	0	8.54	0
Glucose	110.93	10.04	4.08	0
Galactose	14.67	0.72	0	0
Mannose	11.54	10.94	0	0

Sugar concentrations in mg/l determined from tomato xylem sap or the apoplastic fluid before (− bacteria) or after (+ bacteria) sustaining growth of R. solanacearum for 21 h.

**Table 2:**
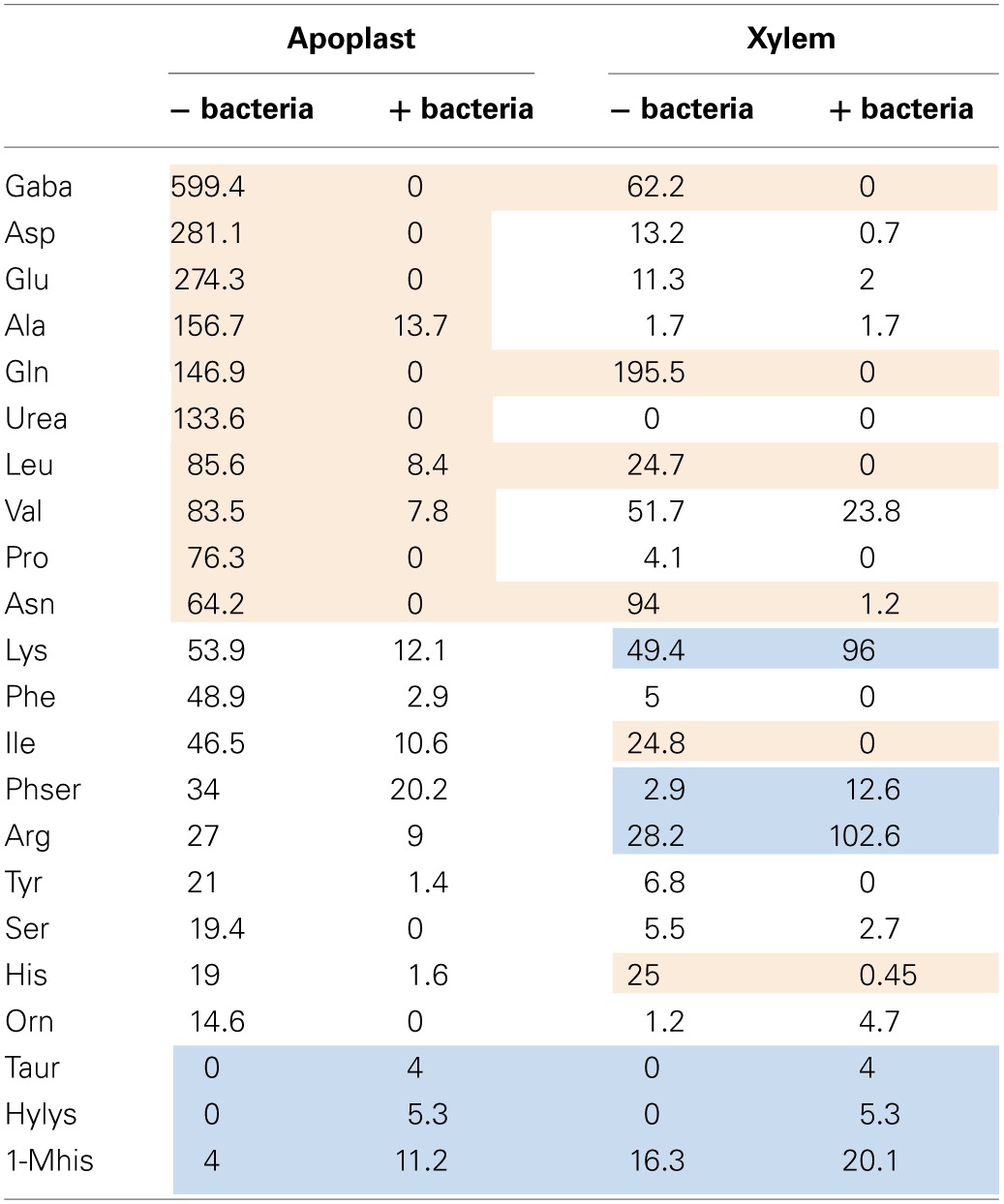
**Aminoacid content in tomato exudates before and after growth of *R. solanacearum***.

Aminoacid concentrations in micromolar units determined from tomato xylem sap or the apoplastic fluid before (− bacteria) or after (+ bacteria) sustaining growth of R. solanacearum for 21 h. Red shading indicates the most abundant aminoacids in each compartment that seem to be metabolized by the pathogen. Blue shading denotes aminoacids whose concentrations increased after bacterial growth. Abbreviations are as follows: Ala, alanine; Arg, arginine; Asn, asparagine; Asp, aspartic acid; Gaba, gamma aminobutyric acid; Glu, glutamic acid; Gln, glutamine; His, histidine; Hylys, hydroxylysine; Ile, isoleucine; Leu, leucine; Lys, lysine; Orn, Ornithine; Phe, phenylalanine; Phser, phosphoserine; Pro, proline; Ser, serine; Taur, taurine; Tyr, tyrosine;Val, valine.

### Plant sugars are readily consumed by *R. solanacearum* during growth in tomato

The possible carbon and energy sources used by *R. solanacearum* during saprophytic growth inside its plant hosts remain unknown. Thus, although we could not identify the *hrpG* inducing signal, the sugar content analyses provided interesting clues on the biology of the bacterium inside the plant. From the results presented in Table 1, it is apparent that the tomato apoplast is rich in sugar contents, in agreement to what has been reported (Rico and Preston, 2008). In addition, we were able to determine that, despite the general assumption that it mainly contains water and minerals, xylem sap was rich in sugars as well. The tomato apoplast showed a high concentration of sucrose, glucose, galactose, mannose and fructose, while the xylem contained glucose and fructose, although at lower concentrations (Table 1). Growth of *R. solanacearum* in these extracts indicated that the bacterium likely catabolizes all abundant apoplast or xylem sugars, which were undetectable after bacterial inoculation. The only exception was the less abundant mannose, which did not seem to be metabolized by bacteria growing in the apoplast and was undetected in the xylem sap (Table 1). Interestingly, studies of two different *R. solanacearum* lectins (RSL) showed contrasting affinity to sugars. RSL was found to bind fucose and arabinose in a higher degree than mannose (Sudakevitz et al., 2002) while RS-IIL recognizes fucose, but displays a higher affinity to fructose and mannose (Sudakevitz et al., 2004). A variable pattern of carbon source utilization correlating with genomic variability has been reported among *Pseudomonas spp* (Rico and Preston, 2008). It would be interesting to test if such metabolic diversity is present also in the *R. solanacearum* species complex and if it correlates with sugar abundance in different plant hosts.

### Aminoacid contents *in planta* vary in different tomato compartments and after bacterial infection

Interesting conclusions could also be extracted from the analyses of the amino acid content and concentrations measured in apoplast and xylem fluids before and after bacterial growth. Table 2 shows all aminoacids and related compounds detected in both apoplast and xylem, ordered from the most to the less abundant in the apoplast. Similar to what had been reported previously (Rico and Preston, 2008), the most abundant amino acid in this tomato intercellular compartment was gamma aminobutyric acid (GABA), followed by aspartic and glutamic acids (Table 2, 1st column). It was also apparent that the xylem presented a very different amino acid composition from the apoplast, with glutamine as the most abundant aminoacid, followed by asparagine and GABA (Table 2, 3rd column). Marked differences in abundance were detected for urea, which was undetectable in xylem but highly abundant in the apoplast and aspartic acid, glutamic acid and alanine, major components of the apoplast that were much less abundant in the xylem (Table 2). These results demonstrated the variability of resources present in plant compartments and suggest that *R. solanacearum* has to cope with these contrasting environments and switch its metabolism along the plant colonization process.

Bacterial growth also impacted the amino acid composition of plant fluids *in vitro*. The amino acid profiles could be divided into those whose concentrations increased after bacterial growth (Table 2, blue shading) and the rest, which often decreased after bacterial growth. Interestingly, the most abundant amino acids were depleted in both compartments after bacterial growth (Table 2, red shading), suggesting bacterial adaptation to preferentially use the most abundant carbon sources. Arginine and lysine are the major exceptions to this rule, since their concentrations were high but almost always increased after bacterial growth in our *in vitro* experiments. Other amino acids whose abundance increased by bacterial metabolism were the least abundant in plant extracts, in agreement with the idea that they do not play a role in pathogen growth (Table 2, blue shading). Interestingly, higher amounts of aminoacids seemed to be released by bacteria in the xylem than in the apoplast. In particular, lysine, arginine and ornithine, which were abundantly produced by the bacteria in the xylem but naturally present in the apoplast and rather consumed by the pathogen growing in this fluid (Table 2). It was previously reported that tryptophan, phenylalanine, tyrosine, leucine, valine, and GABA concentrations increased when *R. solanacearum* grew on tobacco and tomato xylem 3–5 days after inoculation (Coplin et al., 1974). Likewise, Ward et al. (2010) recently described that the levels of tryptophan, tyrosine and phenylalanine increased in abundance in Arabidopsis plants after *Pseudomonas syringae* infection, while sucrose levels decreased. All these aminoacids where found abundantly in our study and rapidly consumed by *R. solanacearum*. We hypothesize that the pathogen might be able to reconfigure the host metabolism to induce the production of the aminoacids required for its growth. Experiments are under way to validate this hypothesis.

## Conclusions

In this work we identified a novel regulatory signal triggering *hrpG* expression at early stages of the interaction with *Arabidopsis* cells. Challenging current knowledge (Aldon et al., 2000; Brencic and Winans, 2005; Rico and Preston, 2008), we show that the transcriptional induction of *hrpG* at early stages of bacterial co-culture with plant cells, which caused overexpression of the downstream T3SS effector genes is independent of bacterial contact with plant cells as demonstrated by the *hrpG* induction in the outer membrane receptor mutant strain *prhA.* The precise plant molecules or environmental cues responsible for the newly described *hrp* gene induction remained unknown. This induction was unaffected after disruption of the known *R. solanacearum* pathogenicity regulators, indicating that it is controlled by a non-described system. Moreover, our work suggests that plant contact-independent signals might also be important *in planta*, as shown by the *hrpG* induction triggered by apoplastic and xylem extracts. However, it must be taken into account that bacterial cultures with either plant cells or plant extracts do not perfectly mimic the spatial and environmental conditions encountered during growth *in planta*. New inputs when bacteria grow parasitically inside the plant host and the real contribution of the signals already described in these natural conditions remain to be determined.

Finally, we gained insight into the plant metabolic resources available for pathogen growth and concluded that invading bacteria not only have to cope with plant defenses but also with contrasting niches inside the host. An example of this adaptation is the specific response of the HrpG virulence regulon to the unknown metabolic or environmental plant signals described here.

## Materials and methods

### Bacterial strains, culture media, and growth conditions

Bacterial strains and plasmids used in this study are listed in Supplementary Table 1. *R. solanacearum* was routinely grown in rich B medium (10 g/l bactopeptone, 1 g/l yeast extract and 1 g/l casamino acids) or Boucher's MM (200 g/l KH_2_PO_4_, 50 g/l (NH4)2SO4, 10 g/l MgSO4-7H2O, KOH 10 N, 1.26 g/l FeSO4,-7H2O) at 30°C. For bacterial growth in plant extracts, 10 ml aliquots of xylem sap or apoplastic fluid in 50 ml erlenmeyers were inoculated with the *R. solanacearum* strain GMI1000 transformed the PhG-lux reporter fusion. The hrpG promoter was PCR-amplified from a cDNA library using primers that added *Avr*II and *Kpn*I restriction sites upstream and downstream of the sequence, respectively. This PCR fragment was cloned into pGEM-T-EASY (Life Technologies, Paisley, UK), giving rise to pG-PhG. The *PhG* promoter was then excised from pG-PhG using *Avr*II-*Kpn*I and cloned into the same sites of pRCG-GWY (Monteiro et al., 2012), creating the plasmid pRCG-PhG-GWY. Finally, to generate pRCG-PhG-lux a *Sfi*I-*Kpn*I fragment containing the entire *LuxCDABE* operon, excised from plasmid pRCGent-Pep-lux (Monteiro et al., 2012), was cloned into the same sites of pRCG-PhG-GWY. This plasmid bears the *PhG::LuxCDABE* reporter fusion and a gentamycin-resistance gene, all flanked by two homology regions for recombination into the bacterial chromosome. Similarly, pRCG-PavrA-lux and pRCG-Pefe-lux were generated by cloning a *Kpn*I/*Bgl*II fragment from plasmid PavrA and pG-Pefe into the same sites of pRCG-GWY, respecively. PCR amplifications were performed with the proofreading Pfx DNA polymerase (Life Technologies, Paisley, UK) following the manufacturer's conditions and other general molecular biology techniques were performed as described in (Ausubel et al., 1994).

### *Arabidopsis* co-culture assays and luminescence measurements

Co-culture expression assays were carried out using *Arabidopsis* cells LT87 grown in Gamborg B5 (GB5). For co-culture assays, bacteria were diluted from overnight cultures grown in B medium to an O.D._600v_= 0.1 in 20 ml-cultures of seven day-old *Arabidopsis* culture cells. Samples were taken every hour to measure cell density and luminescence. To carry out 24 h time course experiments, two cultures were started with 10 h of delay and the results of the two cultures superimposed. To recover only bacterial cells grown in the presence of *Arabidopsis* cells, 1 ml aliquots of the co-cultures were filtrated through a 20 μm-pore nylon membrane as described before (Monteiro et al., 2012). Luminescence measurements of filtered bacteria were done with a Berthold FB-12 luminometer and promoter output of the reporter was expressed as relative luminescence units (RLU) referred to cell density estimated as the O.D._600_ in a Shimadzu UV-1603 spectrophotometer.

### Apoplast and xylem extractions

Apoplast extraction was carried out as described in (Rico and Preston, 2008). Briefly, tomato leaves were cut, washed with distilled water and dried with a paper towel. Then, one to three leaves were introduced into a 50 ml syringe with 20 ml of distilled water and pressure—vacuum cycles applied until the leaves were completely infiltrated. After infiltration, leaves were carefully removed from the syringe and blotted with a paper towel. Each leaf was then introduced into a 5 ml tip placed inside a 50 ml conical tube containing a 1.5 ml collection tube. Apoplast extract was collected by spinning the tubes at 0.6 g for 5 min at 4°C. The fraction collected in the 1.5 ml tube was centrifuged again for 10 min at 0.8 g at 4°C. The supernatant was collected and stored in at −20^°^C until used.

Xylem sap extraction was performed as described by (Kehr and Rep, 2007). Briefly, 5 week-old tomato plants were cut at the stems ~10 cm above ground with a razor blade. The cut stem was rinsed with 2 ml of distilled water and dried with a paper towel to remove the content from cut cells and the first exuded sap. Xylem sap spontaneously oozing from the stem was recovered after discarding the first two drops. Collection tubes were placed on ice and the sap collected for up to 6 h. Xylem sap was filtered through a 0.2 μm pore filter and kept frozen at −20^°^C until used.

### Analysis of aminoacid and sugar contents

Apoplastic and xylem samples (10 ml each) both before and after bacterial growth, were analyzed for their amino acid content by cation exchange chromatography at the Scientific and Technologic Centers from the University of Barcelona (CCITUB). The internal control (norleucine) was added to apoplast and xylem samples and then dried. Samples were resuspended in lithium citrate buffer at pH2.2 and then filtered and injected into the chromatography system (50–100 μl). An automated aminoacid autoanalyzer (Biochrom 30) was used. For sugar quantification, samples (100 μl) were filtered and injected into Aminex HPX-87P (300 × 7.8 mm) + Aminex HPX-87C (300 × 7.8 mm) (BioRad) serial columns in a Waters 717 plus autosampler chromatograph: refractive index Water 2414 at 37°C and *S* = 256. Bacterial content from apoplast and xylem was filtered using a Whatman FP 30/0.45 CA-S pore size 0.45 μm.

## Author contributions

A. Paola Zuluaga performed the experiments, contributed to the experimental design, data analysis, data interpretation, manuscript writing and critical revision. Marina Puigvert performed the experiments and contributed with data analysis and interpretation. Marc Valls contributed to the experimental design, data analysis, data interpretation, drafting of the article and manuscript writing and critical revision.

## Conflict of interest statement

The authors declare that the research was conducted in the absence of any commercial or financial relationships that could be construed as a potential conflict of interest.

## Supplementary material

Table S1 **Description of mutant strains used in this work.** All strains were kindly provided by Dr. S.Genin and transformed with the hrpG-lux in this work. Sp stands for streptomycin/spectinomycin resistance.
