# Supplementary material for: Novel plant inputs influencing Ralstonia solanacearum during infection
Source: Front Microbiol. 2013 Nov 20;4:349. doi: 10.3389/fmicb.2013.00349 (PMC3834233; doi:10.3389/fmicb.2013.00349)
Supplement: Table S1 — Description of mutant strains used in this work. All strains were kindly provided by Dr. S.Genin and transformed with the hrpG-lux in this work. Sp stands for streptomycin/spectinomycin resistance. [file DataSheet1.PDF]

Supplementary Table 1. Description of mutant strains used in this work. All strains were kindly provided by Dr. S.Genin and transformed with the hrpG-lux in this work. Sp stands for streptomycin/spectinomycin resistance.

Strain	Mutation	Resistance	Reference
GMI1575	*prhA*	Sp	Marenda et al (1998)
GMI1579	*prhJ*	Sp	Brito et al (1999)
GMI1580	*prhI*	Sp	Brito et al (1999)
GMI1755	*hrpG*	None	Valls et al (2006)
GRS445	*prhG*	None	Plener et al (2010)
GRS551	*solR*	Sp	Unpublished
GRS574	*xpsR*	Sp	Unpublished
GMI1610	*phcA*	Sp	Genin et al (2005)
GRS573	*vsrA*	Sp	Unpublished
GRS567	*pehR*	Sp	Unpublished
GRS772	*vsrC*	None	Unpublished
